# Left ventricular non-compaction cardiomyopathy in adults – characterisation by cardiac magnetic resonance imaging

**DOI:** 10.1186/1532-429X-11-S1-P242

**Published:** 2009-01-28

**Authors:** Constanze Merten, Stephanie Lehrke, Dirk Lossnitzer, Evangelos Giannitsis, Hugo A Katus, Henning Steen

**Affiliations:** University Hospital Heidelberg, Heidelberg, Germany

**Keywords:** Cardiac Magnetic Resonance Imaging, Impaired Left Ventricular Function, Delay Contrast Enhancement, Enddiastolic Volume, Lateral Left Ventricular Wall

## Introduction

Left ventricular non-compaction (LVNC) is a congenital cardiomyopathy characterised by left ventricular hypertrabecularisation and thinning of the compacted myocardial layer. Due to its high spatial resolution and precise delineation of the non-compacted layer, cardiac magnetic resonance imaging (cMRI) is a promising method to image and diagnose LVNC cardiomyopathy.

## Methods

In 19 patients who underwent cMRI for suspected cardiomyopathy LVNC was diagnosed according to diagnostic criteria such as a ratio of non-compacted to compacted myocardium (NC/C-ratio) of >2.3 in diastole [1] and exclusion of other underlying cardiac pathology or cardiovascular risk factors. We performed cMRI using a clinical 1.5 Tesla scanner (Philips Achieva). Left ventricular (LV) function, dimensions and volumes were assessed using steady-state free precession cine imaging. The NC/C-ratio was assessed in the lateral LV wall in an apical, midventricular and basal short-axis slice. Additionally, delayed contrast enhancement (DCE) imaging was performed.

## Results

Of the 19 patients, 6 were women (32%), the mean age was 41.7 ± 16.8 years. Over the whole study group, LV function was impaired with a mean ejection fraction of 41 ± 17%. However, LV ejection fraction was normal in 6 patients (32%). LV volumes were increased with a mean enddiastolic volume of 247 ± 100 ml and a mean endsystolic volume of 155 ± 99 ml, resulting in mean stroke volume of 92 ± 31 ml. Using the 17-segment model, we found a mean number of 11.8 ± 2.6 segments to be affected by LVNC with the typical predomination of the apical segments and the free lateral LV wall. The mean NC/C-ratio was 5.0 ± 1.6 in the apical lateral wall, 3.3 ± 0.7 in the midventricular lateral LV wall and 2.0 ± 1.1 in the basal lateral wall; the basal septum was involved in none of the patients.

DCE was present in 7 patients (37%); in all patients non-ischemic type DCE with an intramural or spotted enhancement pattern and a predilection for the inferior right ventricular insertion zone was observed.

## Conclusion

We characterised 19 patients with LVNC cardiomyopathy by cMRI. We observed an impaired LV function in the study cohort along with a dilatation of the left ventricle. The mean number of affected segments was 12 of 17. Apical and lateral segments were most frequently affected. Accordingly, the NC/C-ratio decreased from the apical to the basal segments. Non-ischemic type DCE was observed in 37% of patients.
